# Needle-Free Electroacupuncture for Postoperative Pain Management

**DOI:** 10.1155/2011/696754

**Published:** 2011-06-02

**Authors:** Daniel Lee, Hong Xu, Jaung-Geng Lin, Kerry Watson, Rick Sai Chuen Wu, Kuen-Bao Chen

**Affiliations:** ^1^School of Biomedical and Health Sciences, Victoria University, Melbourne, VIC 8001, Australia; ^2^Department of Anesthesiology, China Medical University Hospital, Taichung 40447, Taiwan

## Abstract

This study examined the effects of needle-free electroacupuncture, at ST36 on postoperative pain following hysterectomy. Based on a double-blind, sham and different intervention controlled clinical experimental design, 47 women were randomly allocated to four different groups. Except for those in the control group (Group 1, *n* = 13), a course of treatment was given of either sham (Group 2, *n* = 12), high-frequency stimulation (Group 3, *n* = 12), or low-frequency stimulation (Group 4, *n* = 10). All groups were assessed during the postoperative period for 24 hours. The Visual Analogue Scale was used to determine the amount of perceived pain felt by each subject. Differences were found between the means postoperatively at three, four, eight, 16 and 24 hours. Post hoc comparison tests indicated that Group 4 was significantly different from Groups 1, 2, and 3 at 24 hours. A one-way ANOVA analysis for total patient-controlled analgesia demand and doses indicated significant differences between the groups *F*(3, 42) = 3.59, *P* < .05. Post hoc analysis confirmed the differences between Groups 1 (*M* = 84.54) and 4 (*M* = 41.60). Treatment outcomes of this therapy showed a positive effect for the management of postoperative pain.

## 1. Introduction

Acupuncture has been used for pain relief for thousands of years, and more recently has included electroanalgesic therapies as an alternative to conventional pharmaceutical pain relievers [1]. The aims of this study were to examine the effects of needle-free electroacupuncture (EA) analgesia on subjects recovering from hysterectomies. Silver Spike Point (SSP) needle-free EA was used in this study as opposed to traditional acupuncture, where needles are inserted into acupuncture points. SSP needle-free EA stimulation is achieved continuously with controlled current and frequency ensuring that each subject received the same stimulation and dosages. Xu et al.'s study in Australia [2] confirmed the significant pain-relieving effects that SSP needle-free EA could provide. SSP needle-free EA, if effective, would have the advantage of providing pain control with no opioid-related side effects, such as nausea, dizziness, or the more serious respiratory depression, central nervous system depression including somnolence and conscious disturbance. In this study, it is anticipated that the effects of SSP needle-free EA analgesia on subjects recovering from hysterectomies would be greater than the control groups. The results may help patients by contributing to the knowledge of pain control and providing significant improvement in treatment.

## 2. Methodology

### 2.1. Study Design

Based on a double-blind, sham, and different intervention controlled clinical experimental design. Hysterectomized patients at the China Medical University Hospital were invited to be subjects in the study. The study was approved by the related institutions, and ethics approval was gained from the Human Research Ethics Committee of La Trobe University Australia and China Medical University Hospital Taiwan, before conducting the clinical trial. All subjects were asked to complete a medical suitability form for SSP needle-free EA treatment, received an explanation form, and were asked to sign a consent form. Forty-nine hysterectomized patients who had all received general anesthetics of propofol 2 mg/kg and atracurium 0.5 mg/kg for induction (but no narcotics), and sevoflurane with 50% nitrous oxide and oxygen for anesthesia were randomly allocated to receive one of the four pre- and postoperative procedures. All subjects were randomly assigned to the four groups, and the group assignments were kept fully blinded from the subjects and data collectors. 

Neither the subjects nor the research assistant carrying out the procedures was aware of who was receiving the therapy. The subjects were treated in silence in the surgery preparing room before the operation. The chief of the anesthetic team in the operation theatre marked the point on the subjects' legs. Neither the doctor performing the interventions, the subjects, nor the nurse collecting the results was aware of the differences between the different interventions. Therefore, there was no disclosure to the subjects in relation to what interventions they had received. 

In this study there were four groups of subjects (Table 1). To ensure that the groups consisted of randomly assigned subjects, each subject was assigned to a treatment group in numbered order, as they became available. A random number table was used for grouping. Every subject in each group received patient-controlled analgesia (PCA) (Pain Management Provider TM, Abbott Laboratories, North Chicago, IL, USA). The groups differed only in the acupuncture procedure they received. In an attempt to control for the placebo effect of SSP therapy, one “sham acupuncture” treatment group was included. 

According to Traditional Chinese Medicine (TCM) theory, the acupuncture point Zusanli (ST36) was selected for this study, as it lies on the stomach meridian that traverses through the abdominal area surrounding the female reproductive organs. 

In Groups 2, 3, and 4, subjects received treatment commencing 30 minutes before general anesthesia. Subjects in Group 2 received no stimulation. Subjects in Group 1 received general anesthesia only. Group 3 received SSP stimulation at 100 Hz, and Group 4 received SSP stimulation by a mixture of dense-sparse wave at 3 Hz, 10 Hz, and 20 Hz. After surgery, SSP needle-free EA treatment was administered once only and was initiated once the subject regained consciousness in the recovery room. The SSP needle-free EA procedure was provided for 30 minutes, the 1/f Yuragi waves were used. All subjects in each group received PCA postoperatively. As the subjects received their treatment in different rooms, confidentiality was strictly followed, and as a result there was no opportunity for them to communicate with each other. Furthermore, as the subjects had no prior experience of such treatment they were unable to identify which treatment they are receiving. 

All groups were assessed during the postoperative period for 24 hours using a Visual Analogue Scale (VAS), and total amount of PCA demand and total amount of PCA doses were recorded. The time of the first PCA demand and the frequency of subsequent demands were noted over the first 24-hour period postoperatively. The total dosage of morphine used was calculated for each group. A VAS measurement for pain intensity was administered. The data was collected over a 24-hour period commencing postoperatively (see Figure 2). 

Before discharge, the subjects were asked to answer questions pertaining to the occurrence of any side effects from the SSP needle-free EA treatment, such as bruising, dizziness, and anxiety. The VAS was again used to determine the intensity of pain.

### 2.2. Subjects

Subjects with medical conditions such as hypertension, diabetes, cachexia, cardiac, respiratory, kidney and nervous system problems, coagulopathy, and other bleeding disorders were excluded. Subjects with a history of opioid use and sensitivity to opioid-related side effects (e.g., nausea, vomiting) were also excluded. Each subject was instructed on the operational aspects of the PCA device at the initial preoperative visit and again in the recovery room. The use of SSP needle-free EA was also explained to Groups 2, 3, and 4 before the operation.

The real acupuncture point ST36 is on the lower leg, three units (approximately the patient's four fingers width) inferior of the knee patellar ligament, and approximately one finger width lateral to the anterior crest of the tibia. The sham acupuncture point (on the same level as ST36 but above the tuberositas tibia) was identified in all subjects one day prior to the operation. Subjects received one of four different pre- and postoperative treatment regimes. All subjects were completely anesthetized throughout the surgery. After surgery, subjects were transported to postanesthetic recovery (PAR).

### 2.3. Measurements

The analgesic dosage levels at which patients experience pain relief varies greatly as does the patient's sensitivity to pain. Using PCA, patients are able to determine their own analgesic requirements. After one hour in the recovery room, all subjects in the four groups were connected to a PCA system providing IV morphine with boluses of 0.8 mg in the subsequent 23 hours. There was a lockout time of 8 minutes. Doses were registered on a chart. Immediately on return to the general ward from the recovery room, the subjects were asked to complete a VAS for intensity of pain at the time. The VAS is a 10 cm line with extreme limits marked with perpendicular lines and appropriate labels. Point zero on the line equals no pain, and the other end point, 10 cm away, equals maximum pain. There are no words or numbers at the end points. The subject is requested to mark the line with an X reflecting their level of pain by the appointed nurse. The main advantage of this scale is that it can be viewed as a ratio scale with an infinite number of points on the line. 

The time of the first requirement of morphine, the postoperative analgesic requirement (in the 24 hours), and the number of PCA demands (i.e., button presses) in the first 24 hours were recorded. The subject's age, weight, the duration of anesthesia and surgery, first requirement of morphine, VAS scores, and PCA demands were analyzed using one-way analysis of variance, comparing the difference between groups of control, sham acupuncture, high-frequency stimulation, and low-frequency stimulation.

## 3. Results

**Figure 1:**
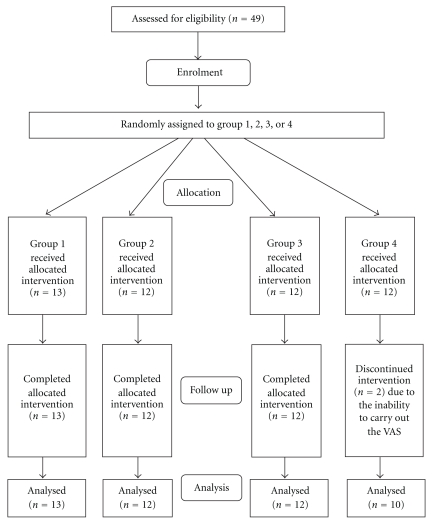
Flow chart of participants through each stage of the trial.

Forty-seven (47) subjects who had hysterectomies were finally included in the study (Figure 1). The results were analyzed using a one-way analysis of variance (ANOVA) for VAS, PCA doses delivered, and PCA doses demanded by the subjects. This method is for testing the differences between the means of independent samples. In this case, using SPSS and Student-Newman-Keuls (SNK) and Tukey post hoc analysis performed a one-way ANOVA. *F*-tests were also carried out to determine significance between means of the variables (*M*), time of first ambulation and bowel movement, total PCA demand, and total PCA doses. *F* distributions are given with degree of freedom of 3 and 42 or 3 and 43.

### 3.1. Subjects Description

Forty-nine (49) consenting females undergoing a hysterectomy were involved in the study. Two of the subjects from Group 4 were excluded from the analysis due to the inability to carry out the VAS. The mean age ± standard deviation (SD) of the 47 subjects was 42.02 ± 8.31 years. The mean duration ± SD of the operation was 127.70 ± 34.56 minutes with an anesthetic time 166.04 ± 35.95 minutes (Table 2).

### 3.2. Postoperation General Recovering Conditions

Subjects' related general recovering conditions, that is, time of first bowel movement, time of first ambulation (when patients felt they were capable of ambulating), and opioid-related effects after the operation were recorded, and comparisons were made between groups using *F*-tests, with degree of freedom of 3 and 42 or 3 and 43. 

Table 3 shows the mean times (hours) and SD of the first bowel movement after the hysterectomy for subjects within the four groups.  Table 4 shows the mean times (hours) and SD of first ambulation after the hysterectomy for subjects within the four groups. As can be seen in Tables 3 and 4, the means and SD of the four groups are within a close range. *F*-tests were performed on all means for each group for both time of first ambulation and time of first bowel movement, and no significant differences between the means were found.

### 3.3. Visual Analogue Scale

**Figure 2:**
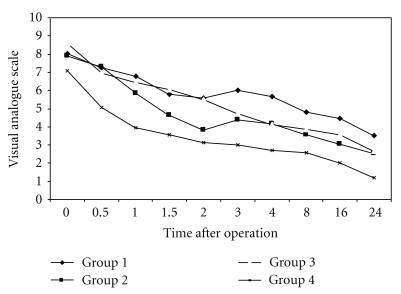
Pain perceived by subjects over a 24-hour period using the visual analogue scale.

The VAS was used to determine the amount of perceived pain felt by subject (Table 5). The data was collected over a 24-hour period commencing postoperatively. A one-way ANOVA was used to test for significant differences between the groups at each specific time interval. Figure 2 shows a clearly decreasing trend in the amount of pain felt over the 24-hour period for all the four groups with respect to the PCA and time. 

Differences were found between the means at two hours postoperatively, *F*(3,42) = 2.66, *P* < .10; three hours postoperatively, *F*(3,42) = 3.68, *P* < .05; four hours postoperatively, *F*(3,42) = 4.33, *P* < .05; eight hours postoperatively, *F*(3,42) = 3.33, *P* < .05; sixteen hours postoperatively, *F*(3,42) = 4.25, *P* < .05; twenty-four hours, *F*(3,42) = 4.67, *P* < .01. Further post hoc analysis showed that at one hour postoperatively, Groups 1 and 4 were different to each other at the *P* < .10 level. Also at three hours, Group 1 (*M* = 6.05) was significantly higher, at the *P* < .05 level, than Group 4 (*M* = 3.00). This difference was also found at four hours, Group 1 (*M* = 5.65) and Group 4 (*M* = 2.71); eight hours, Group 1 (*M* = 4.81) and Group 4 (*M* = 2.57); sixteen hours, Group 1 (*M* = 4.46) and Group 4 (*M* = 2.00); twenty-four hours, Group1 (*M* = 3.50) and Group 4 (*M* = 1.21). *M* corresponds to the mean values as given in Table 5.

Both post-hoc comparison tests indicate that Group 4 was significantly different from Groups 1, 2, and 3 at twenty-four hours.

### 3.4. Patient-Controlled Analgesia Doses

A one-way ANOVA was applied to determine the differences between the means over the four time intervals for PCA doses requirement (Table 6).

**Figure 3:**
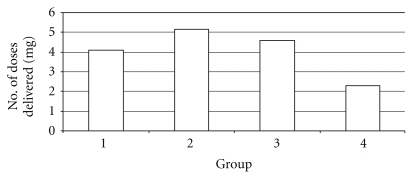
PCA doses delivered in the postoperative room.

Significant differences were found, *F*(3,43) = 3.69, *P* < .05, in the postoperative room (POR) between the groups. Post-hoc analysis confirmed and found that Group 4 (*M* = 2.30) was significantly lower than Groups 2 (*M* = 5.17) and 3 (*M* = 4.58). Post hoc comparison tests indicate that mean PCA doses in the POR for Group 4 was significantly different from Groups 2 and 3. Mean PCA doses between Groups 1 and 4 showed no differences. These differences are demonstrated in Table 6 and Figure 3. Differences were also found between the groups between one and eight hours postoperatively, *F*(3,43) = 2.33, *P* < .10. However, post hoc analysis did not confirm this finding between any of the four groups.

### 3.5. Patient-Controlled Analgesia Doses Demanded

Table 7 shows that the four groups have a similar trend within 24 hours postoperatively. However, significant differences were found between the means at the POR, *F*(3,43) = 4.80, *P* < .01, and one to eight hours postoperatively, *F*(3,43) = 3.49, *P* < .05. Post hoc analysis confirmed the significant difference between the means at one to eight hours postoperatively that Group 2 (*M* = 55.75) was significantly higher than Group 4 (*M* = 22.30). 

Post hoc analysis also confirmed that Group 2 (*M* = 30.83) was significantly higher than Group 3 (*M* = 13.00) and Group 4 (*M* = 5.90) at the POR. No other significance was found between means.

### 3.6. Total Patient-Controlled Analgesia Doses and Demands

As demonstrated in Table 8, there was no significant difference between the total PCA doses times given for the four groups over 24-hour period postoperatively. However, a one way ANOVA was applied to compare the groups for total PCA doses demanded, and significant differences were found between the groups *F*(3,42) = 3.59, *P* < .05. Post hoc analysis confirmed the difference between Groups 1 (*M* = 84.54) and 4 (*M* = 41.60). Moreover, differences were found between the four groups for the amount of analgesia delivered in mg, *F*(3,43) = 2.45, *P* < .10. Post-hoc analysis confirmed this finding between Groups 1 (*M* = 38.63) and 4 (*M* = 29.29).

## 4. Discussion

### 4.1. Subject Number

In this study the initial plan was to recruit 60 subjects as a clinical trial; however, 49 subjects enrolled in this study and achieved significant outcomes in several measurements. Other studies which produced significant results show that subject numbers vary in many pain management projects using acupuncture, EA or SSP. Kurokawa [3] reported that a clinical trial involving 30 subjects (10 in each group) showed positive outcomes of using SSP. Ishimaru et al. [4] indicated that significant results were gained by using EA in 16 subjects. 

### 4.2. Postoperation General Conditions

The point ST36 is expected to regulate bowel movement, relieve abdominal pain, and improve energy [5] and, therefore, improve the time of subject's first bowel movement and time of first ambulation and reduce opioid-related effects after the surgery. The fact that there was no significant difference between the groups with regard to first bowel movement time and first ambulation time is a point which awaits to be studied.

### 4.3. Total PCA Demand and Doses

A similar study conducted by Lin [6], explored the effects of high- and low-frequency electroacupuncture in pain after lower abdominal surgery. The results showed that the first time of analgesic request was 10, 18, 28, and 28 minutes in the control, sham-, low-, and high-EA groups, respectively. In addition, during the first 24 hours 21, 43, and 61% in the sham-, low-, and high-EA groups decreased the total amount of morphine required, respectively. 

In this study, the results showed that the PCA doses delivered in the postoperative room were 4.08, 5.17, 4.58, and 2.30 mg in the control, sham-, high-, and low-EA groups, respectively. However, the total amount of dose delivery during the first 24 hours decreased to 53, 43, 53, and 48% in the control, sham-, high-, and low-EA groups, respectively.

### 4.4. Pain Intensity

The results of this study showed that there were significantly lower mean pain scores in the treatment groups compared with the control group. Pain assessment, for the first two hours, was every half an hour, and subsequently at the 3rd, 4th, 8th, 16th, and 24th hours by VAS. All groups had high mean pain scores at zero hour. In Groups 1 and 4, the difference was significant at the 1st, 3rd, and 4th hours. However, in Groups 1 and 3, the significant difference was shown at the 2nd hour and parallel to the 24th hour. It seems that SSP needle-free EA can help with postoperative pain management. While the positive pain-reducing effects are evident within the measured time frame, further investigations involving the long-term treatment effects may be worthy of future study.

The positive pain-relieving results found in this study could be supported by the theory that endorphins may be produced by both SSP electrode stimulation and acupuncture. Alternatively when Naloxone, an antinarcotic substance, is administered, it is found to reverse the analgesic effect of endorphins as it is able to combine with morphine receptors stronger than endorphins [7]. The results of the study may be interpreted that low-frequency surface point stimulation such as SSP is also related to endorphin release in the body.

## 5. Conclusion

Silver spike point needle-free electroacupuncture has shown to have positive effects for postoperative pain management. The findings of this study are important for those patients who seek nonpharmacological analgesia without side effects. Although further study is needed to ultimately determine whether SSP needle-free EA has a place in postoperative pain treatment, this study shows that SSP needle-free EA may be able to make a contribution as an adjunct to other forms of medical intervention. Arguably this “needle-free approach” has benefits for patients who have difficulties with traditional acupuncture needling and at the same time are interested in a nonpharmacological approach to treat pain.

## Figures and Tables

**Table 1:** The proposed four experiment groups and their treatment regimes.

Subject groups	EA treatment	
	Preoperative	Postoperative
Group 1 PCA	NIL (control group)	NIL (control group)
Group 2 PCA	SSP electrode at sham acupuncture point near to ST36 (no stimulation) for 30 minutes.	SSP electrode at sham acupuncture point near to ST36 (no stimulation) for 30 minutes.
Group 3 PCA	SSP electrode at ST36 with continuous wave of 100 Hz electrical stimulation for 30 minutes.	SSP electrode at ST36 with continuous wave of 100 Hz electrical stimulation for 30 minutes.
Group 4 PCA	SSP electrode at ST36 with dense-sparse wave 3 Hz/4 sec, 10 Hz/4 sec, and 20 Hz/4 sec electrical stimulation for 30 minutes.	SSP electrode at ST36 with dense-sparse wave 3 Hz/4 sec, 10 Hz/4 sec, and 20 Hz/4 sec electrical stimulation for 30 minutes.

**Table 2:** Description of subjects.

	*N*	Minimum	Maximum	Mean	SD
Age (years old)	47	14.00	59.00	42.02	8.31
Weight (Kg)	47	40.00	158.00	59.74	17.54
Duration (min)	47	65.00	240.00	127.70	34.56
Anesthesia time (min)	47	94.00	275.00	166.04	35.95
Valid *N *	47				

**Table 3:** Time of first bowel movement (in hours).

Group	Number	Mean	SD
1	13	37.54	2.93
2	12	38.03	2.71
3	12	35.50	2.64
4	10	33.30	2.43

**Table 4:** Time of first ambulation (in hours).

Group	Number	Mean	SD
1	13	32.50	2.41
2	12	33.75	3.11
3	12	33.13	3.18
4	10	29.90	2.95

**Table 5:** Pain perceived by subjects over a 24-hour period using the visual analogue scale (in rank 0–10).

Gr.	Time after operation (hours)									
	Zero	Half	One	One-half	Two	Three	Four	Eight	Sixteen	Twenty-four
1	8.04 ± 2.65	7.27 ± 2.34	6.77 ± 2.29	5.81 ± 2.19	5.58 ± 2.13	6.00 ± 1.57	5.65 ± 1.56	4.81 ± 1.38	4.46 ± 1.38	3.50 ± 1.27
2	7.92 ± 2.26	7.29 ± 2.19	5.83 ± 1.98	4.63 ± 1.69	3.83 ± 1.39	4.38 ± 1.61	4.17 ± 1.70	3.54 ± 1.54	3.04 ± 1.20	2.54 ± 0.86
3	8.59 ± 1.71	6.95 ± 2.65	6.45 ± 2.78	6.05 ± 3.03	5.48 ± 3.02	4.73 ± 2.30	4.14 ± 2.11	3.86 ± 1.71	3.55 ± 1.67	2.68 ± 1.63
4	7.07 ± 3.44	5.07 ± 3.62	3.93 ± 2.98	3.57 ± 2.96	3.14 ± 2.30	3.00 ± 2.27^∧^	2.71 ± 1.83^∧^	2.57 ± 1.41^∧^	2.00 ± 1.75^∧^	1.21 ± 1.31^∧^

^∧^ *P* < .05.

**Table 6:** Dose of patient-controlled analgesia delivered (in mg).

Group	Means over the four time intervals for PCA doses delivered			
	POR	One–eight hours	Eight–sixteen hours	Sixteen–twenty four hours
1	4.08 ± 1.66	18.08 ± 5.64	10.88 ± 4.50	9.67 ± 3.30
2	5.17 ± 2.98	17.73 ± 3.52	8.28 ± 2.50	7.55 ± 2.37
3	4.58 ± 1.31	15.34 ± 2.80	7.68 ± 3.55	8.13 ± 4.66
4	2.30 ± 2.00^∧^	14.22 ± 3.55	8.30 ± 3.67	6.77 ± 3.43

^∧^ *P* < .05.

**Table 7:** Means of patient-controlled analgesia demanded by subjects of 24 hours postoperatively (in times of requests).

Group	Means of patient-controlled analgesia demanded by subjects of 24 hours postoperatively			
	POR	One–eight hours	Eight–sixteen hours	Sixteen–twenty four hours
1	15.08 ± 11.34	49.54 ± 34.61	22.08 ± 14.03	12.92 ± 6.06
2	30.83 ± 28.98	55.75 ± 36.76	13.08 ± 10.18	8.75 ± 6.44
3	13.00 ± 4.95	29.75 ± 13.21	12.25 ± 9.20	10.17 ± 10.43
4	5.90 ± 4.32^∧∧^	22.30 ± 19.90^∧^	11.80 ± 12.41	7.50 ± 5.37

^∧^ *P* < .05; ^∧∧^ *P* < .01.

**Table 8:** Total PCA demanded and delivered (in mg).

Group	Total PCA doses given for the four groups over 24-hour period postoperatively		
	Times delivered 24 hr	Times demand 24 hr	Dosage delivered 24 hr
1	29.16 ± 11.79	84.54 ± 38.66	38.63 ± 11.08
2	31.82 ± 11.98	74.18 ± 39.33	34.20 ± 6.40
3	27.75 ± 11.78	52.17 ± 21.87	31.14 ± 8.15
4	23.30 ± 11.93	41.60 ± 36.47	29.29 ± 8.89^∧^

^∧^ *P* < .05.
